# Neural Oscillation During Mental Imagery in Sport: An Olympic Sailor Case Study

**DOI:** 10.3389/fnhum.2021.669422

**Published:** 2021-06-01

**Authors:** Dagmara Budnik-Przybylska, Adrian Kastrau, Patryk Jasik, Maria Kaźmierczak, Łukasz Doliński, Paweł Syty, Marta Łabuda, Jacek Przybylski, Selenia di Fronso, Maurizio Bertollo

**Affiliations:** ^1^Department of Sport Psychology, Institute of Psychology, Faculty of Social Science, University of Gdańsk, Gdańsk, Poland; ^2^Department of Theoretical Physics and Quantum Information, Institute of Physics and Computer Science, Faculty of Applied Physics and Mathematics, Gdańsk University of Technology, Gdańsk, Poland; ^3^Department of Family Studies and Quality of Life, Institute of Psychology, Faculty of Social Sciences, University of Gdańsk, Gdańsk, Poland; ^4^Department of Mechatronics and High Voltage Engineering, Faculty of Electrical and Control Engineering, Gdańsk University of Technology, Gdańsk, Poland; ^5^BioTechMed Center, Gdańsk University of Technology, Gdańsk, Poland; ^6^Department of Medicine and Aging Sciences, Behavioral Imaging and Neural Dynamics (BIND) Center, University “G. d’Annunzio” of Chieti-Pescara, Chieti, Italy

**Keywords:** imagery, EEG, flow state, attentional focus, neural efficiency, transient hypofrontality hypothesis, alpha, athlete

## Abstract

The purpose of the current study was to examine the cortical correlates of imagery depending on instructional modality (guided vs. self-produced) using various sports-related scripts. According to the expert-performance approach, we took an idiosyncratic perspective analyzing the mental imagery of an experienced two-time Olympic athlete to verify whether different instructional modalities of imagery (i.e., guided vs. self-produced) and different scripts (e.g., training or competition environment) could differently involve brain activity. The subject listened to each previously recorded script taken from two existing questionnaires concerning imagery ability in sport and then was asked to imagine the scene for a minute. During the task, brain waves were monitored using EEG (32-channel g. Nautilus). Our findings indicate that guided imagery might induce higher high alpha and SMR (usually associated with selective attention), whereas self-produced imagery might facilitate higher low alpha (associated with global resting state and relaxation). Results are discussed in light of the neural efficiency hypothesis as a marker of optimal performance and transient hypofrontality as a marker of flow state. Practical mental training recommendations are presented.

## Introduction

Imagery is a multisensorial mental representation of the image of actions (or objects and situations), without an actual experience and without appropriate sensory input (White and Hardy, 1998; Kosslyn, 2005). Images could be recalled from memory or could be a novel combination of stimuli (Pearson et al., 2015). Imagery can serve cognitive and motivation functions and each at a general or specific level (Paivio, 1985; Hall et al., 1998). In the sports domain, mental training techniques including imagery serve to improve athletes’ optimal performance (Morris et al., 2005; Cumming and Williams, 2012; Munroe-Chandler and Guerrero, 2017), which has been usually associated with the flow state (Csikszentmihalyi, 1990; Jackson and Roberts, 1992; Jackson et al., 1998, 2001).

Therefore, during competition athletes can experience various mental states, even when performing optimally, in different conditions and with a different level of cognitive control (Bortoli et al., 2012; Robazza et al., 2016; Ruiz et al., 2020). These multi-states were also associated with the underpinning cortical activity (Bertollo et al., 2016; di Fronso et al., 2016). For instance, in the multi-action plan (MAP) model (Bortoli et al., 2012), the flow like states (i.e., automatic optimal performance—Type 1) have been associated with the overall cortical synchronization on the task in line with the neural efficiency theory as suggested by Del Percio et al. (2009) and Hatfield (2018). In shooting it is likely related to a “default mode network” functioning, proper autonomous skills, and goal-relevant attentional focus when approaching shot release.

Research has indicated an enhanced flow induced by imagery interventions in the context of sports training and competition (Jeong, 2012; Koehn et al., 2014) due to athletes’ clear focus on task execution without experiencing emotions (Koehn et al., 2016). For instance, Koehn and Díaz-Ocejo (2016) confirmed an increase in flow state in runners with individually adjusted imagery scripts after 4 weeks of intervention (see for an overview on the use of imagery in endurance McCormick et al., 2019). Indeed, there is evidence that individually-adjusted scripts are more efficient and meaningful for the athletes’ imagery (Williams et al., 2013); moreover, a higher psychophysiological activity is observed when the scripts are prepared by participants rather than by experimenters (Wilson et al., 2010). Additionally, self-produced imagery can provide useful information on the athlete’s understanding of the imagined scene by activating their own personal mental representations (Lindsay et al., 2020).

Therefore, imagery scripts should be tailored for specific aims, adjusted to the level of the athlete (Williams et al., 2013), and should induce not only flow like states (i.e., Type 1 performance states) but also functional-controlled states (i.e., Type 2 performance; see MAP model for the type of performance; Bertollo et al., 2016, 2020). Moreover, personalized scripts can stimulate mental representation of the action which is based on Bio-informational theory (Lang, 1985) but also the individual’s cognitive representation of an action (Frank et al., 2014). Thus, differently from earlier studies at the neural level which involved simple movements and motor imagery, in the present study, we used personalized and whole scripts.

### Flow and Superior Human Performance in Sport Neuroscience

The experience of flow state is characterized by automatic information processing without conscious thinking and by the temporary suppression of prefrontal cortex (PFC) activity- which is in accordance with the *transient hypofrontality hypothesis* (Dietrich, 2003, 2004; Dietrich and Audiffren, 2011). Moreover, in the sports domain, it is observed that elite athletes, in line with the *neural efficiency* hypothesis present more efficient task-related neural networks. It means that neural activity is reduced in experts, which might suggest that elite athletes’ brain is characterized by more efficient resources distribution, more economic activity or hypoactivation (Haier et al., 1992; Hatfield and Kerick, 2007; Del Percio et al., 2009; Duru and Assem, 2018).

Studies on superior human performance reported low alpha event-related desynchronization (ERD) in elite athletes, for example in expert golfers when the stroke was correct compared to a missed one (Babiloni et al., 2008) or in elite gymnasts judging sports actions (Babiloni et al., 2009). Similarly, elite shooters presented high alpha (about 10–12 Hz) event related synchronization (ERS) rather for high score shots than for low score shots, mainly in right parietal and left central areas (Del Percio et al., 2009). Additionally, flow states were associated with increased theta activities in the frontal areas and moderate alpha activities in the frontal and central areas (Katahira et al., 2018), which was explained by a higher level of cognitive control and immersion in the task, and a lower level of working memory loading. Moreover, Wolf et al. (2015), while analyzing EEG signals, revealed that elite table tennis athletes experiencing flow during imagery present reduced influence of verbal-analytic processing on motor control. This is reflected in the deactivation (higher alpha power) of a left temporal site (T3), connected to verbal-analytic processing when compared to the opposite right site (T4) associated with visuospatial processing.

Research on comparisons of brain activation in expert vs. novice athletes indicated that alpha ERD values were lower in experts (e.g., in expert shooters; Haufler et al., 2000). Additionally, elite athletes compared to amateur athletes and non-athletes presented greater resting alpha power, which was explained by higher inhibition of the athlete’s brain during resting states (Babiloni et al., 2010). In the context of a flow, Wolf et al. (2015) suggested that elite table tennis players as compared to novice athletes presented lower left-temporal brain activity and a significantly stronger right temporal-premotor coherence. Moreover, the stronger the right hemisphere activation, the more the athlete experienced the flow state. The right hemisphere was probably activated during complex visuomotor performance (Rebert et al., 1984).

Not considering imagery, other research concerning neural efficiency in sport has linked low energy costs with simultaneous high-level performance (Del Percio et al., 2008, 2009; Percio et al., 2010). In this context, di Fronso et al. (2016) have identified some of the neural markers underlying optimal and suboptimal performance of an elite air-pistol shooter. ERD-ERS analysis supported the notion that optimal-automatic performance experiences were characterized by a global ERS of cortical arousal associated with the shooting task, whereas suboptimal controlled states were underpinned by high cortical activity levels in the attentional brain network. These findings echoed that a high-level athlete’s performance is associated with reduced cortical activity (Bertollo et al., 2016).

### Mental and Motor Imagery in Neuroscience

Over the last years, there has been an increase in research on imagery that involves the analysis of psychophysiological parameters and neuroimaging techniques (Hardwick et al., 2018). Such studies refer to the fact that movements, regardless of whether they are real or imagined, stimulate the same neurons, engage comparable patterns of connectivity between cortical motor regions (Gao et al., 2011) and similar trails of the movement are stored in the memory (Jeannerod, 1994; Munzert and Zentgraf, 2009; Guillot et al., 2012; Hétu et al., 2013; Hardwick et al., 2018).

Mental imagery has been reflected in alpha power, which has been considered as a good indicator of the way the brain works when a mental effort is required (Cremades, 2016). It has been associated with the alpha activity from the parietal (somatosensory representation of the skin, senses, and kinesthesia) and occipital (visual processing) cortical areas (Ray and Cole, 1985; Marks and Isaac, 1995; Cremades and Pease, 2007; Cremades et al., 2010; Pearson et al., 2015). Xie et al. (2020) found that representation of visual imagery and perception shared similarity in the alpha frequency band in the parieto-occipital cortex. The electro-cortical differences following audio or visual instructions used in guided kinesthetic and visual imagery have been confirmed by Cremades (2016). Imagery performance induced greater power in lower and upper alpha at the right hemisphere than the left. Specifically, greater lower and upper alpha values were detected at the temporal site of the right hemisphere. Spagna and coworkers (Spagna et al., 2021) discovered that visual mental imagery engages the left fusiform gyrus, and patients with extensive left temporal damage often have this type of imagery impairment. But speech imagery blocks the alpha and theta band in the left hemispheric frontal and temporal regions (Chengaiyan et al., 2018).

Hardwick et al. (2018) in their meta-analysis of the brain correlates of motor imagery, observation, and execution of movements found that motor imagery and action observation recruited similar premotor-parietal cortical networks. Moreover, motor imagery also recruited a subcortical network similar to movement execution. Specifically, motor imagery primarily recruited a network of bilateral premotor, rostral inferior and middle superior parietal, basal ganglia, and cerebellar regions, with left-lateralized recruitment of the dorsolateral prefrontal cortex. Other studies on brain correlates of motor imagery were mainly focused on mu rhythm in association with the imagination of simple movements or showed that motor imagery was mainly characterized by alpha ERD (Cebolla et al., 2015, 2017).

In the present study, we refer to guided imagery interventions (vs. self-produced imagery) that involve scripts to follow whilst focusing and directing participants’ imagination. In medicine guided imagery serves to empower patients to actively participate in their recovery or control their emotional reactions (Hadjibalassi et al., 2018). Imagery guided visually or by using audio stimuli might facilitate creating mental images that bring about a state of focused concentration, which in turn, allows for relaxation (Tusek et al., 1997). As compared to imposed imagery scripts self-produced imagery generates a higher psychophysiological activity in electromyography as observed by Wilson et al. (2010) who found greater activity in the right bicep when the participants created their own scripts. Moreover, this kind of imagery can provide useful information of the athlete’s understanding of the imagined scene by activating their own personal mental representation (Lindsay et al., 2020).

### Aim of the Current Study

The purpose of the current study was to examine the cortical correlates of imagery depending on instructional modality (guided vs. self-produced) using various scripts related to training and competition involving all sensory systems, emotions, and feelings. Mental imagery implemented as a part of sports training should: (a) activate all senses (White and Hardy, 1998; Holmes and Collins, 2001; Morris et al., 2005); (b) produce similar reactions; and (c) activate similar brain regions to the real situation (Hardwick et al., 2018). Additionally, peak performance should be associated with flow state and thus involve reduced activity in brain regions linked to self-referential processing which are related to relaxation (stress management) along with an enhanced focus on the performed task (Csikszentmihalyi, 1997; Bertollo et al., 2016). Earlier research has indicated that relaxation states were associated with low alpha, especially in the left hemisphere. On the contrary, the attentional focus was linked to higher alpha (Hatfield and Kerick, 2007; Bertollo et al., 2020). Therefore, it is well described in the literature that elite athletes display various emotions and mental states during their performance (see Hanin, 2000). According to the expert-performance (Ericsson, 2020) approach we took an idiosyncratic perspective analyzing the mental imagery of an experienced two-time Olympic athlete to verify whether different instructional modalities of imagery and different scripts (e.g., training or competition environment) could differently involve brain activity assuming that neural efficiency is a marker of optimal performance and transient hypofrontality is a marker of flow state.

We hypothesized the following effects of the two modes of inducing images:

H1: Neural oscillation associated with self-produced imagery.

H1a: general higher power in low alpha as compared to guided imagery.
H1b: higher power in low alpha in temporal regions of the left hemisphere compared to guided imagery.

H2: Neural oscillation associated with guided imagery.

H2a: general higher power in high alpha and SMR as compared to self-produced imagery.
H2b: higher activation of sensory-motor and frontal regions.
H2c: left prefrontal hypoactivation and concurrent hyperactivation of the left temporoparietal areas.

## Materials and Methods

### Participant

A 32-year-old two-times male Olympic sailor voluntarily agreed to participate in the case study. He is a member of the sailing national team and participated two times in the Olympic Games, as well as in numerous national and international events including the European and World Championships. The athlete was experienced in mental training, including imagery, and used to work with sport psychologists. Indeed, he achieved high scores in imagery ability questionnaires which are presented in detail in the instruments section.

The investigation followed the ethical principles regarding human experiments as defined in the Declaration of Helsinki and the study was approved by the local Institutional Review Board (University of Gdańsk, 11/2015). Written consent was obtained from the athlete to participate in the study. Moreover, written informed consent was obtained from the participant for the publication of any potentially identifiable images or data included in this article.

### Instruments

Two questionnaires to assess the imagery ability in sport-related situations and assessing multiple dimensions of imagery but also general tendency to use imagery were implemented.

The Imagination in Sport Questionnaire (ISQ; Budnik-Przybylska, 2014) is a multidimensional 51-item measure that consists of seven subscales, i.e., physiological feelings (noticeable changes in body functioning), modalities (use of all senses besides the visual), ease/control (ease and control of imagined scene), perspective (juggling of different perspectives of the imagined scene), affirmations (positive attitude during competition), visual (visual sense), and general (general tendency to use imagery). The participant imagined a competitive situation for 60 s in as detailed and realistic manner as possible and then responded to the 51 items rating the different aspects of each image on a scale of 1–5 (located next to each statement by entering the appropriate number, where 1 means “not at all” and 5 “completely so”). In Polish studies, Chronbach’s alpha for the subscales varied between 0.65 and 0.79. The sailor achieved higher scores (i.e., ease/control = 47, perspective = 40, affirm = 39, visual = 30, feelings = 25, modal = 22, general = 27, total = 230) than those achieved by other Polish elite athletes (for means and SD see: Budnik-Przybylska, 2014). The following was the description of the imagined situation:

Start in a high-level championship. Imagine yourself before the start of high-level competitions. Spend about 60 s on this task. If you want, you can close your eyes. Try to keep the image as realistic as possible, have as many details, and pay attention to all the elements. Imagine what you see, what you hear and what you feel, what you’re doing, what others are doing, and what is happening around you. Feel the emotions and sensations that this situation has on you.

The Sport Imagery Ability Measure (SIAM) is a questionnaire developed by Watt and Morris (2001) that consist of 48 items grouped in 12 scales which assesses five imagery dimensions (vividness, control, ease of generation, speed of generation, and duration), six senses (visual, auditory, kinesthetic, olfactory, gustatory, and tactile sense), and the experience of emotions. In previous studies, including the SIAM validation in Polish, the instrument showed concurrent validity with the ISQ, and a moderate to high reliability with a Chronbach’s alpha ranging from 0.73 to 0.90 (Budnik-Przybylska et al., 2014; Budnik-Przybylska, 2014).

Respondents are asked to imagine five scenes: *Fitness activity, Start in a high-level championship, Successful Competition, Training Session, Your Home Venue* (Fitness activity—is the example of the scene not used to calculate the total score) associated with the sports situations. After 60 s of imagining each of the four generic sports scenes, they provide self-report ratings of 48 imagery ability items (12 for each imagined scene), one reflecting each dimension, sense modality, and emotion. At the end of the imagery interval, they are requested to rate each scenario by placing a cross (X) on a 100 mm visual analog scale for each item. Respondents can obtain from 0 to 4,800 points as a total score and from 0 to 400 points for each subscale.

The sailor achieved higher scores than those achieved by athletes from the national level from the Polish sample; for means and SD see Budnik-Przybylska et al. (2014) in the majority of subscales (vividness 394, control 394, ease of generation 397, speed of generation 385, duration 396, auditory 268, visual 393, kinesthetic 391, olfactory 248, gustatory 255, tactile sense 379 and total 4,028), except from the emotion subscale (128), in which the subject scored much lower. Following are the examples of scenes:

Fitness Activity. Imagine yourself doing an activity to improve your fitness for your sport. Get a clear picture of what you are doing, where you are, and who you are with. Take notice of what you can see around you, the sounds you hear, and the feel of any muscles moving. Do you get the sensation of any smells or tastes? Can you feel the equipment and surfaces you are using? Do you get an emotional feeling from this activity? Now you have 60 s to create and experience your image of the scene.

Successful Competition. Imagine you are competing in a specific event or match for your sport. Imagine that you are at the very end of the competition and the result is going to be close. You pull out a sensational move, shot, or effort to win the competition. Take notice of what you can see around you, the sounds you hear, and the feel of any muscles moving. Do you get the sensation of any smells or tastes? Can you feel the equipment and surfaces you are using? Do you get an emotional feeling from this activity? Now you have 60 s to create and experience your image of the scene.

### EEG Recording

The raw electroencephalographic data were recorded using the 32-channels (incl. ground and reference). Nautilus wearable EEG headset with 24-bit ADC accuracy at 250 Hz sampling rate. The instantaneous values in the time domain have been stored for each electrode in the double-precision floating-point format, and then saved in the MATLAB data file. The accompanying data consisted of the recorded markers using a push-button, indicating the beginning of each scene in the time domain. For convenience, that original dataset was then converted into separate CSV files, storing raw data for different scenes according to the markers.

### Procedure

The sport psychologist (first author), who used to work with the athlete, recorded six scripts that were linked to the questionnaires previously described, which were part of the training routine of the sailor. The athlete listened to the recorded scripts (the recorded script lasted from 33 to 47 s), then had a 2-s break for washing out the mind and finally was asked to imagine the scene for a minute. There were the following six scenes in each condition: *Fitness Activity*, *Start in a high-level championship* (precompetitive routine), *Successful Competition*, *Slow Start*, *Training Session*, *Your Home Venue*. Each scenario was first listened to and then imagined with the eyes closed in both conditions.

During these two imagery modalities (listened-guided imagery and imagined-self-produced imagery), the athlete’s brain activity (i.e., neural oscillation) was monitored by the EEG. Between listened-guided imagery and imagined-self-produced imagery of each specific scene, there was a short break (2-s). However, between each of the blocks of scripts there was a longer break to allow the athlete to change his position and take a rest. Additionally, the participant was asked to fill in the imagery questionnaires—The SIAM and the ISQ—to verify his imagery ability.

### EEG Data Processing and Analysis

Studying imagery, there were different analyses of the EEG data that could have been implemented. For instance, following previous studies (e.g., Cebolla et al., 2015, 2017) we could have implemented event-related spectral perturbation (ERSP) comparing the two conditions of imagery with the baseline. However, in the current study we were interested in analyzing the neural oscillation during the two conditions of guided imagery and self-produced imagery and for this reason, we decided to perform a time-frequency analysis using Fast Fourier Transformation (FFT). Now, we describe the preprocessing, processing, and postprocessing data analysis.

### Preprocessing

The EEG signals coming from 30 channels (electrodes) were divided into six situations according to the collected EEG markers and timings of considered situations. Such prepared raw EEG signals were visually inspected to identify improper channels exposing non-physiological behavior, outliers, and unidentified peaks, as well as zero or constant value regions. No bad channels were detected. The next step of the chosen preprocessing procedure was to apply Butterworth’s FIR filter (Butterworth, 1930; Kastrau, 2019) on all EEG signals. Since we were interested in theta, alpha, and beta bands we filtered the data using a Butterworth bandpass filter using an order of the filter, low and high-frequency range as defined in the following formula.

$$f_{\min}=1\;Hz;\;f_{\max}=40\;Hz;\;order=5.$$

Then, we applied the Hjorth method on signals of all electrodes, in order to remove some EEG-related artifacts from the raw signal. The spatial Laplacian filter is successfully used in many signal processing and BCI applications (Kołodziej, 2011; Murugappan, 2011; McFarland, 2015). Here, the small Laplacian filter (McFarland et al., 1997) was used as defined below.

$$X_{new}=X_{old}(t)-\frac{1}{N}\sum_{x=1}^{N}x_{i}(t),$$

where *X*_*new*_ is the transformed signal, *X*_*old*_(*t*) current potential on the electrode, N—number of adjacent electrodes. The new signal is calculated by subtracting the current potential from the averaged potential from adjacent electrodes. In this article, we have calculated the average potential of appropriate nearest neighbors. We also removed the mean values of all channels in order to obtain the stationarity of the EEG signals. Finally, we applied Independent Component Analysis (ICA), implemented in the MNE-Python package (Montoya-Martínez et al., 2017; Ablin et al., 2018; Artoni et al., 2018). The ICA method is a powerful tool in the case of removing various artifacts from the signal, especially ocular artifacts (eye blinks, eye movements). For each situation, we calculated 30 independent components, which were visually inspected to search potential artifacts. We did not observe any components related to the eye movements and blinking to be removed for this athlete. The probable reason for this situation is the closed eyes of a person participating in the experiment.

### Processing

To obtain specific EEG rhythms, we transferred the preprocessed signals of all electrodes from the time domain to the frequency domain. Since the EEG signals are real sequences, we used the function rfft, which computes the 1-D n-point discrete Fourier Transform (DFT) of a real-valued array by means of an efficient algorithm called the Fast Fourier Transform (FFT), as implemented in the Python scipy.fft package using Kaiser windowing (Nunez-Iglesias et al., 2017; Discrete Fourier transforms (scipy.fft), 2021). Then we applied the appropriate bandpass filters which allowed us to extract frequency ranges given in hertz (Hz) units corresponding to considered brainwaves: theta [4, 8], low alpha [8, 10], high alpha [10, 12], alpha [8, 12], SMR [12, 15], low beta [15, 23], high beta [23, 30], and beta [15, 30]. In the next step of the processing procedure, we returned to the time domain by using the irfft function (Discrete Fourier transforms (scipy.fft), 2021), which is the inverse of the 1-D n-point discrete Fourier Transform of real input computed by rfft. Eventually, we obtained the pure brainwaves ready to use in further analysis.

### Postprocessing

For the purposes of the current study, we were interested in low alpha, high alpha, and SMR brainwaves. Therefore, for these EEG rhythms, we split signals into the guided imagery part and the self-produced imagery part according to the timeframes of each considered situation. Then we calculated the mean power of the selected brainwaves for all electrode signals and both parts of each situation. Next, we computed differences between the mean values of the self-produced imagery part and the guided imagery part for all electrodes and all situations. All the above allowed us to create head maps showing the activities of the brain within low alpha, high alpha, and SMR rhythms in the cases of both parts of considered situations as well as differences between them. In order to create head maps more informative, we used MinMax normalization of the mean values of all electrode signals for the self-produced imagery part, guided imagery part, and differences between them for all situations. To have an idea of the neural oscillation in the entire brain we have plotted the head maps using all the electrodes, however, being interested in the hemisphere differences, the statistical analysis was focused on two hemisphere (right and left) and 10 regions of interest (ROIs): frontal right—{Fp2, F4} and left—{Fp1, F3}; central right—{C2, C4, FC2} and left—{C1, C3, FC1}; temporal right—{T8, C6, FC6} and left—{T7, C5, FC5}; central parietal right—{CP2, CP6} and left—{CP1, CP5}; and finally parieto occipital right—{P4, PO8} and left—{P3, PO7}. Therefore, we also calculated the mean power of each ROI and hemisphere for all situations.

### Statistical Analysis

A Wilcoxon signed ranks test was employed to determine significant differences between the two types of instruction in low and high alpha as well as in SMR, according to each script, hemispheres, and regions.

## Results

**Figure 1:**
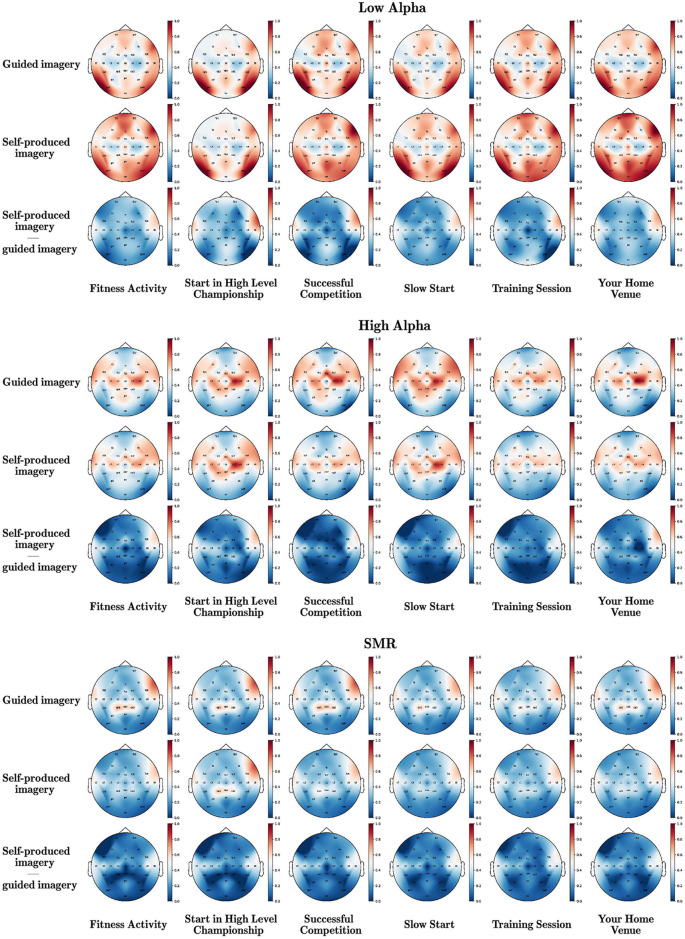
Brain waves underpinning different instructional modalities (guided vs. self-produced) of imagery in various scripts. The mean power values of all electrodes were normalized using the MinMax method and the red color corresponds to 1, while the blue color corresponds to 0.

The head maps of low alpha, high alpha, and SMR during the two imagery instructional modalities (guided vs. self-produced) and the six scripts are displayed in Figure 1, reporting also the difference between the two conditions.

A Wilcoxon-signed-ranks-test indicated that low alpha in self-produced imagery was significantly higher than in guided imagery in almost all scenes (except from successful competition—but the direction was the same). Adversely, high alpha and SMR were significantly higher in guided than in self-produced imagery (significant differences were observed in high alpha in the majority of the scripts except from Start in a high level championship and Your Home Venue, but still the pattern of differences was similar). Detailed statistics are presented in Table 1.

**Table 1:** Differences between guided and self-produced imagery in analyzed situations and brainwaves.

Situation	Wave	Guided imagery		Self-produced imagery		
		*M*	SD	*M*	SD	*Z*
Successful competition	Low alpha	7.02	9.94	7.42	10.39	0.09
	High alpha	3.29	2.80	2.36	1.85	3.30***
	SMR	1.43	1.03	0.93	0.60	2.98**
Fitness activity	Low alpha	6.48	9.14	7.49	10.60	2.67**
	High alpha	3.29	3.10	2.54	2.16	3.11**
	SMR	1.45	1.03	0.80	0.56	3.23**
Slow start	Low alpha	6.37	9.14	7.47	10.69	3.30***
	High alpha	3.62	3.32	2.67	1.83	2.98**
	SMR	1.18	0.80	0.71	0.40	3.30***
Start in a high level championship	Low alpha	6.59	9.92	7.88	10.94	2.79**
	High alpha	3.23	2.67	3.01	2.07	0.03
	SMR	1.32	0.91	0.60	0.30	3.04**
Training session	Low alpha	6.72	9.28	7.64	10.59	2.10*
	High alpha	2.92	2.52	2.06	1.45	3.30***
	SMR	1.28	0.82	0.84	0.65	3.30***
Your home venue	Low alpha	7.03	9.96	8.43	11.89	3.30***
	High alpha	2.66	2.22	2.39	1.75	1.29
	SMR	1.37	1.01	0.81	0.52	3.23**

*p < 0.05, **p < 0.01, ***p < 0.001.

A Wilcoxon-signed-ranks-test indicated that SMR in guided imagery was significantly higher than in self-produced imagery in both hemispheres and all regions (except from the central region in the left hemisphere, however, the pattern of changes was in the same direction). A similar tendency was observed in the right hemisphere for high alpha in the following brain regions: frontal, central, and temporal. As concerns, the left hemisphere, high alpha in guided imagery compared to self-produced imagery was significantly higher in the frontal, temporal, and central parietal regions. However, low alpha in self-produced imagery was significantly higher in the frontal and temporal brain regions in the left hemisphere. Although the main focus was on the differences between left and right hemispheres, we also observed that guided imagery was higher compared to self-produced in the SMR in the CPz (*M* guided imagery = 0.59, SD = 0.19; *M* self-produced = 0.32; SD = 0.08; *Z* = 2.20; *p* < 0.05). Detailed statistics are presented in Table 2.

**Table 2:** Differences between guided and self-produced imagery in brain regions and brainwaves.

			Guided imagery		Self-produced imagery		
Hemisphere	Region	Wave	*M*	SD	*M*	SD	*Z*
Right	Frontal	Low alpha	4.20	1.09	4.94	0.49	1.69
		High alpha	2.24	0.46	1.84	0.35	2.03*
		SMR	1.42	0.31	0.87	0.21	2.37*
	Central	Low alpha	1.39	0.12	1.50	0.10	1.52
		High alpha	4.33	0.33	3.51	0.36	2.37*
		SMR	0.99	0.17	0.80	0.09	2.37*
	Temporal	Low alpha	3.34	0.68	3.95	0.32	1.35
		High alpha	2.19	0.19	1.71	0.38	2.37*
		SMR	1.27	0.46	0.60	0.07	2.37*
	Central_parietal	Low alpha	4.31	0.37	4.48	0.29	0.68
		High alpha	2.10	0.56	1.79	0.54	1.69
		SMR	1.60	0.32	0.89	0.21	2.20*
	Parieto_occipital	Low alpha	32.14	2.14	34.66	2.19	1.86
		High alpha	6.39	1.71	5.25	0.73	1.86
		SMR	3.32	0.43	2.11	0.54	2.37*
Left	Frontal	Low alpha	3.47	0.47	4.29	0.35	2.20*
		High alpha	1.68	0.28	1.37	0.31	2.03*
		SMR	0.96	0.16	0.62	0.07	2.37*
	Central	Low alpha	1.14	0.18	1.18	0.12	0.68
		High alpha	4.00	0.66	3.34	0.40	1.69
		SMR	0.56	0.05	0.58	0.11	0.17
	Temporal	Low alpha	1.62	0.17	1.94	0.19	2.20*
		High alpha	1.84	0.20	1.51	0.27	2.03*
		SMR	0.92	0.26	0.55	0.08	2.37*
	Central_parietal	Low alpha	3.70	0.33	3.96	0.20	1.35
		High alpha	1.74	0.36	1.46	0.34	2.20*
		SMR	1.36	0.30	0.76	0.16	2.20*
	Parieto_Occipital	Low alpha	17.42	0.76	17.90	1.20	0.85
		High alpha	4.26	0.86	3.72	0.50	1.35
		SMR	2.15	0.25	1.43	0.29	2.20*

*p < 0.05.

## Discussion

The main aim of our study was to examine the neural oscillation underpinning the two modalities of imagery (guided and self-produced) during various scenes in a 32-year-old two-times male Olympic-sailor. We observed that in accordance with the hypothesis H1a low alpha was significantly higher in self-produced imagery as compared to guided imagery in almost all imagined scripts. The achieved results are in line with the previous studies concerning elite athletes, in which experts have generally presented an increased oscillation of the low alpha associated with a lower energy expenditure according to the neural and psychomotor efficiency (Haier et al., 1992; Hatfield and Kerick, 2007; Del Percio et al., 2009; Duru and Assem, 2018). On the contrary, in accordance with the hypothesis H2a, high alpha, and SMR were significantly higher in guided imagery in most of the scripts. Indeed, the above results indicate that guided imagery instructional modality induces stronger attentional focus (Tusek et al., 1997; Hadjibalassi et al., 2018) and verbal-analytical processing during conscious motor control with higher high alpha and SMR, whereas self-produced imagery instructional modality might facilitate higher relaxation with higher power in low alpha. These findings are also generally consistent with the idea that a relaxed and/or well-focused athlete exhibits a strong alpha oscillation before the accomplishment of a motor task (Cheron et al., 2016).

Self-produced imagery (as compared to guided) instructional modality induced more low-alpha in the frontal and temporal left hemisphere, which meant that the right hemisphere was more “active”. We also observed higher power of low alpha in the temporal regions of the left hemisphere which confirmed the reduced verbal analytic processing in flow (Wolf et al., 2015). Thus, the hypothesis H1b is confirmed.

However, we observed higher power of high alpha in the left hemisphere during guided imagery than during self-produced, which means higher activation of the left hemisphere and more attentional focus. Our results are in line with Hatfield and Kerick’s Kerick’s (2007) study, in which left- temporal/parietal regions were activated during phonological analysis and homologous right parts of the brain were linked to visual-spatial processes. Therefore, we could confirm our expectation. Overall, these findings are also consistent with the notion that alpha oscillation can reflect the inhibition of the cortex in order to exert cognitive control on the performance (e.g., Klimesch et al., 2007; Cheron et al., 2016).

While taking brain regions and hemispheres under consideration our results indicated that guided imagery (as compared to self-produced) instructional modality activated more frontal and temporal regions in both hemispheres, central-right regions, and centro-parietal regions in the left hemispheres (high alpha and SMR). We also observed a lower power of low alpha in the both frontal regions and in the temporal left region, which confirmed our hypothesis H2b and H2c. These results are also consistent with the notion that various regions of the brain are highly intercorrelated (neural network) and one domain can influence another (Hatfield and Kerick, 2007). While the frontal region was more active during guided imagery than in self-produced imagery, it was still less active than other brain regions, which might be explained by the transient hypofrontality hypothesis (Dietrich, 2003, 2004; Dietrich and Audiffren, 2011) as far as inhibition of activation within the prefrontal cortex (PFC) is concerned. Our results are also in line with the study of Leroy and Cheron (2020) who found a lack of frontal activity during flow conditions in the EEG recording of a tightrope professional in real action. Thus, imagery enhanced the automaticity of the subject’s performance characteristic for the flow state (Harris et al., 2017). We also observed the higher activation of the central region in guided imagery, which may be explained by higher activation of sensory-motor regions of the brain, and the representation of the imagined movement (Hardwick et al., 2018), which is also confirmed by the SMR oscillation.

From a practical standpoint, our findings suggest that guided imagery instructional modality should be implemented in athletes’ training routines to enhance mental effort and to control emotions (e.g., fear; Paivio, 1985; Martin et al., 1999). In consequence, the controlled state (e.g., Type 2) performance in the MAP model (Bertollo et al., 2016; di Fronso et al., 2016) might be achieved, and it could involve an action-centered coping strategy (Hanin and Hanina, 2009). In that way, our sailor might have identified any component of his behavior (e.g., balanced leading of the boat and staying calm) as essential for his attention and the improved execution of the task. On the contrary, self-produced imagery instructional modality might be used to achieve the Type 1 performance (Bertollo et al., 2016; di Fronso et al., 2016). In consequence, the athlete performs in the optimal but automatic way, linked to a flow-like attentional mode (Robazza et al., 2016). We might conclude that, in the context of elite athletes or high efficacy in sports performance and high emotional regulation abilities, cortical activity can also be an expression of attentional focus and mental effort during imagery.

The present study is not free from limitations. Although this is a case study, according to the suggestion made by Ericsson (2020), the investigation of an elite expert athlete provides a lot of useful information that cannot be collected from general athletes. Our study was the first, in which the whole imagery scripts were taken under consideration as opposed to analyses of simple movements or only one kind of imagery (i.e., motor imagery) presented in earlier studies. However, the complexity of the scripts might have compromised the strength of the evidence of the presented analyses and thus conclusions drawn from this study. The next limitation of the study design came from the fact that the subject had his eyes closed, which generated more alpha waves in the occipital region. Still, while comparing brain activity for the two instructional modalities we discovered significant differences in SMR (with significant decreases during self-produced imagery), which means that the subject probably used motor imagery (Niedermeyer and Lopes da Silva, 2004). Another limitation is that we concentrated only on the alpha and SMR bands and future research could also investigate theta and beta bands with different hypotheses.

## Conclusions

This study examined the cortical correlates of imagery depending on two instructional modalities (guided vs. self-produced) using various sport-related scripts. Such a methodological approach enabled us to confirm that instructional imagery modality facilitates cortical activity which can be an expression of attentional focus and mental effort during imagery. Thus, our results confirmed that imagery mental training might still be performed and developed to achieve a particular mental state, even when considering elite-level athletes. Moreover, the obtained results not only confirm but also elaborate on the transient hypofrontality and neural efficiency hypotheses.

## Data Availability Statement

The datasets presented in this study can be found in online repositories. The names of the repository/repositories and accession number(s) can be found below: the data that support the findings of this study are available from the corresponding author upon reasonable request and are openly available in MOST Wiedzy at https://mostwiedzy.pl/en/open-research-data-series/niema,202103272042247055361-0/catalog.

## Ethics Statement

The studies involving human participants were reviewed and approved by University of Gdańsk Ethics committee (University of Gdańsk, 11/2015). The patients/participants provided their written informed consent to participate in this study. Written informed consent was obtained from the participant for the publication of any potentially identifiable images or data included in this article.

## Author Contributions

DB-P designed the study of the project, cooperated with the participant, contributed to the acquisition, analysis, interpretation of data as well as wrote the original draft of the manuscript. AK, PJ, ŁD, and PS contributed to the analysis, interpretation of data, and concept and design of the project. MK contributed to the analysis, interpretation of data, drafting of the manuscript, and concept and design of the project. JP contributed to the acquisition and analysis. MŁ and SF contributed to the concept and design of the project, review, and editing of the manuscript. MB contributed to the analysis, interpretation of data, drafting of the manuscript, concept and design of the project, and supervising EEG data analysis and interpretation. All authors contributed to the article and approved the submitted version.

## Conflict of Interest

The authors declare that the research was conducted in the absence of any commercial or financial relationships that could be construed as a potential conflict of interest.
